# Assessing AAV stability on affinity resins and the effects of long contact times through extensive washing

**DOI:** 10.3389/fbioe.2026.1954356

**Published:** 2026-10-30

**Authors:** Lukas Bongers, Leon Clemenz, Jorge Navas Martinez, Melanie Weber, Jürgen Hubbuch, Roberto Falkenstein, Andres D. Martinez

**Affiliations:** 1 Gene Therapy Technical Research & Development, Roche Diagnostics GmbH, Penzberg, Germany; 2 Department of Biomolecular Separation Engineering, Karlsruhe Institute of Technology, Karlsruhe, Germany

**Keywords:** adeno-associated viral vector, affinity chromatography, critical quality attributes, direct loading, downstream processing, gene therapy, impurity removal, transduction efficiency

## Abstract

Affinity chromatography represents the standard platform for the capture of recombinant adeno-associated viral vectors (rAAVs). However, low upstream titers frequently necessitate long processing time exceeding multiple hours, the biophysical and functional consequences of which remain largely uncharacterized. Here, we present an extensive dataset characterizing the effects of long capsid-ligand interaction times on rAAV critical quality attributes. We examined the impact of binding durations (0–24 h) at room temperature *versus* 10 °C, using three different serotypes (rAAV2, AAV8 wild-type (WT), AAV9WT) and several affinity ligands, all based on single-domain V_H_H-fragments (POROS™ CaptureSelect™ AAVX, POROS™ CaptureSelect™ AAV9, Capto™ AVB). Using CaptureSelect AAVX, extended hold times ( ≥ 8 h) resulted in serotype-specific yield losses correlated with shifts in capsid viral protein (VP)-ratios, alongside enhanced host-cell impurity clearance, without compromising transgene integrity. Washing for 8 h or longer reduced elution yields by 28% for AAV9 and 35% for rAAV2, whereas AAV8 yield remained unaffected. This yield loss coincided with an increased relative abundance of VP3 for rAAV2 (+1.5% at 8 h), while AAV8 exhibited no changes in VP-ratio. Crucially, host-cell protein clearance was substantially improved across all serotypes, with log removal factor increasing from 3.34 to 4.71 for AAV8, 3.34 to 4.87 for rAAV2, and 2.77 to 4.91 for AAV9. LC-MS-based proteomics revealed that DNA-interacting proteins, known to co-elute with rAAVs during affinity chromatography, were cleared more than 10-fold during several hour-long binding times compared to standard processes.

## 1 Introduction

*In vivo* gene therapies promise sustained clinical efficacy after a single administration (Mendell et al., 2017; Dunbar et al., 2018). Treatments targeting various rare genetic disorders have been developed, including voretigene neparvovec-rzyl (Luxturna) for retinal dystrophy, onasemnogene abeparvovec (Zolgensma) for spinal muscular atrophy and etranacogene dezaparvovec (Hemgenix) for Hemophilia B, all of which utilize adeno-associated viral vectors (AAV) as gene delivery vehicle (Mullard, 2023). AAVs are small, non-enveloped capsids belonging to the *Dependoparvovirus* genus within the *Parvoviridae* family, typically measuring approximately 25–30 nm in diameter (Agbandje-McKenna and Kleinschmidt, 2011). These vectors are preferred in clinical applications due to their high tissue tropism, low human immunogenicity and their ability to transduce both dividing and non-dividing cells (Calcedo and Wilson, 2013; Srivastava, 2016).

Thirteen wild-type (WT) AAV serotypes have been characterized (Mietzsch et al., 2021), additionally numerous recombinant AAV (rAAV) constructs have been engineered to address clinical requirements, such as enhanced tissue tropism and improved transduction efficacy (Li et al., 2025; Büning and Srivastava, 2019). Across all serotypes, AAVs share a conserved icosahedral (*T* = 1) surface structure, featuring cylindrical channels at the five-fold axes involved with active DNA packaging, protrusions at the three-fold axes and depressions at the two-fold axes (Agbandje-McKenna and Kleinschmidt, 2011). The capsid is composed of 60 viral proteins (VP) in three isoforms, VP1, VP2 and VP3. VP3 is the most abundant and structurally conserved subunit, VP1 and VP2 contain unstructured N-terminal extensions that are located within the interior of a capsid (Govindasamy et al., 2006; Nam et al., 2007; Xie et al., 2002), (Kronenberg et al., 2005; Bleker et al., 2005). Although a stoichiometric ratio of 1:1:10 is frequently reported, recent stochastic models indicate that AAV populations are highly heterogenous, with the single most abundant capsid composition representing less than 2.5% of the total capsid population (Wörner et al., 2021). Post-translational modifications (PTMs) occurring during vector production are recognized as additional contributors towards AAV heterogeneity. Modifications such as deamidation, phosphorylation, acetylation and glycosylation can alter the charge of affected residues, resulting in a broad distribution of surface charges across the vector population (Mary et al., 2019; Giles et al., 2018).

AAV capsids are susceptible to several physical degradation pathways including aggregation, genome ejection and transgene fragmentation. Stress can be caused by various conditions such as freeze/thaw cycles, low ionic strength or extreme pH with effects strongly depending on the construct (Wright et al., 2005; Burns and Datta, 2025; Heckel et al., 2026). Structural heterogeneity further complicates this landscape, as subpopulations may react differently to environmental stressors (Khanal et al., 2023). Consequently, downstream purification processes must maintain the structural integrity of a heterogeneous population while removing host cell impurities, aggregated viral particles and capsids with absent, fragmented, partial or non-effective transgene. Liquid chromatography provides a scalable, good manufacturing practice (GMP)-compliant solution. Standard protocols typically employ an initial affinity chromatography step featuring single-domain V_H_H-fragments or peptides, followed by anion exchange chromatography (AEX) for the enrichment of full capsids (Jungbauer and Wheelwright, 2025).

Current large-scale manufacturing primarily relies on camelid-derived single domain antibody ligands, favored for their high dynamic binding capacities exceeding 5x10^14^ capsids/mL of resin (Laroudie and Bazot, 2024). Serotype-agnostic affinity ligands such as POROS™ CaptureSelect™ AAVX (AAVX) or Capto™ AVB (CaptoAVB) and serotype-specific ligands like POROS™ CaptureSelect™ AAV9 (CSAL9) target highly conserved regions around the 5-fold pore (Mietzsch et al., 2024; Mietzsch et al., 2020) and require acidic pH for capsid elution (Terova et al., 2018). However, the long binding durations caused by low upstream titers raise significant stability concerns. Khanal et al. (Khanal et al., 2023) demonstrated that extended retention duration (up to 8 h) on strong AEX resins can alter capsid elution profiles and increase the binding strength of empty capsids, thereby compromising the resolution between full and empty capsids. Furthermore, they reported evidence of on-column degradation, correlating capsid stability with transgene presence (Khanal et al., 2023). Although transient triple transfection remains the predominant method for vector production, ongoing upstream developments are heavily focused on engineering stable producer cell lines to overcome these inherent titer limitations and improve scalability (Fu et al., 2026; Stewart et al., 2010). Until stable cell lines achieve widespread commercial adoption, downstream operations must continue to accommodate the lower yields of triple-transfection processes. Given that these processes yield titers in the range of 10^12^ capsids/mL (Nie et al., 2025), the duration for which capsids remain bound to affinity ligands during loading can significantly exceed the timeframes previously studied for AEX. For instance, assuming a residence time of 3 min (Chaubal et al., 2026), achieving a target load density of 5x10^14^ capsids/mL would require a loading phase of approximately 25 h. As affinity chromatography remains standard for AAV downstream processing, any process-induced alterations to product quality introduced by this purification step must be systematically characterized and monitored. To address this gap, the effects of long binding times (up to 24 h) to affinity resins were investigated for three AAV constructs (rAAV2, AAV8WT, AAV9WT).

In this study, we conducted a systematic assessment of several physical, chemical and biological characteristics including capsid and viral genome titers, post-translational modifications (PTMs), aggregation, impurity clearance and transduction efficiency. For experimental consistency, previously concentrated material was loaded within 10 min. Continuous loading of AAVs in a dynamic flow system was then mimicked by flowing equilibration buffer with comparable pH, conductivity and additives to that of the AAV feedstock for several timeframes up to 24 h. This approach was further validated for AAV8WT by directly comparing the effects of rapid, high concentration loading against a 24-h loading period using unconcentrated material. To distinguish ligand-binding mediated stresses from potential load material instability and to investigate stress prior to loading, capsid stability was additionally analyzed under static on-bench conditions. By evaluating this extensive dataset across multiple AAV serotypes and affinity resins, we aim to provide detailed product understanding during purification processes. Ultimately, these insights offer a framework for robust process design, ensuring the structural and functional integrity of AAV-based gene therapies throughout manufacturing.

## 2 Materials and methods

### 2.1 Material production

The serotypes investigated in this study include non-replication-competent wild-type AAV8 (AAV8WT), wild-type AAV9 (AAV9WT) and a recombinant AAV2 (rAAV2) variant. Each construct carried an identical 4.7 kb transgene encoding the fluorescent reporter mGreenLantern (mGL). Vector stocks were produced in-house *via* a standardized 500 L scale triple-transfection process utilizing HEK293 cells, as previously described (Bongers et al., 2026). The harvested cell culture fluid was subsequently concentrated 15-fold *via* tangential flow filtration (TFF). This ultrafiltration step was performed using an ÄKTA readyflux™ XL system (Cytiva, Uppsala, Sweden) equipped with TangenX® SIUS® TFF cassettes (Repligen, Waltham, MA, USA), maintained at a feed pressure of 1.0 bar and a controlled transmembrane pressure (TMP) of 0.6 bar. Following concentration, resulting titers were in the range of 10^13^ capsids/mL, with full-to-total ratios spanning 18%–25%. Pools were aliquoted and frozen at −80 °C for subsequent chromatography experiments.

### 2.2 Experimental setup

To evaluate the stability of AAVs during long binding times to affinity ligands, we performed affinity chromatography operations in which capsids were quickly loaded and then washed for up to 24 h before elution. The elution pools were collected and analyzed further. Experimental details can be found in Figure 1 and are additionally described below:

**FIGURE 1:**
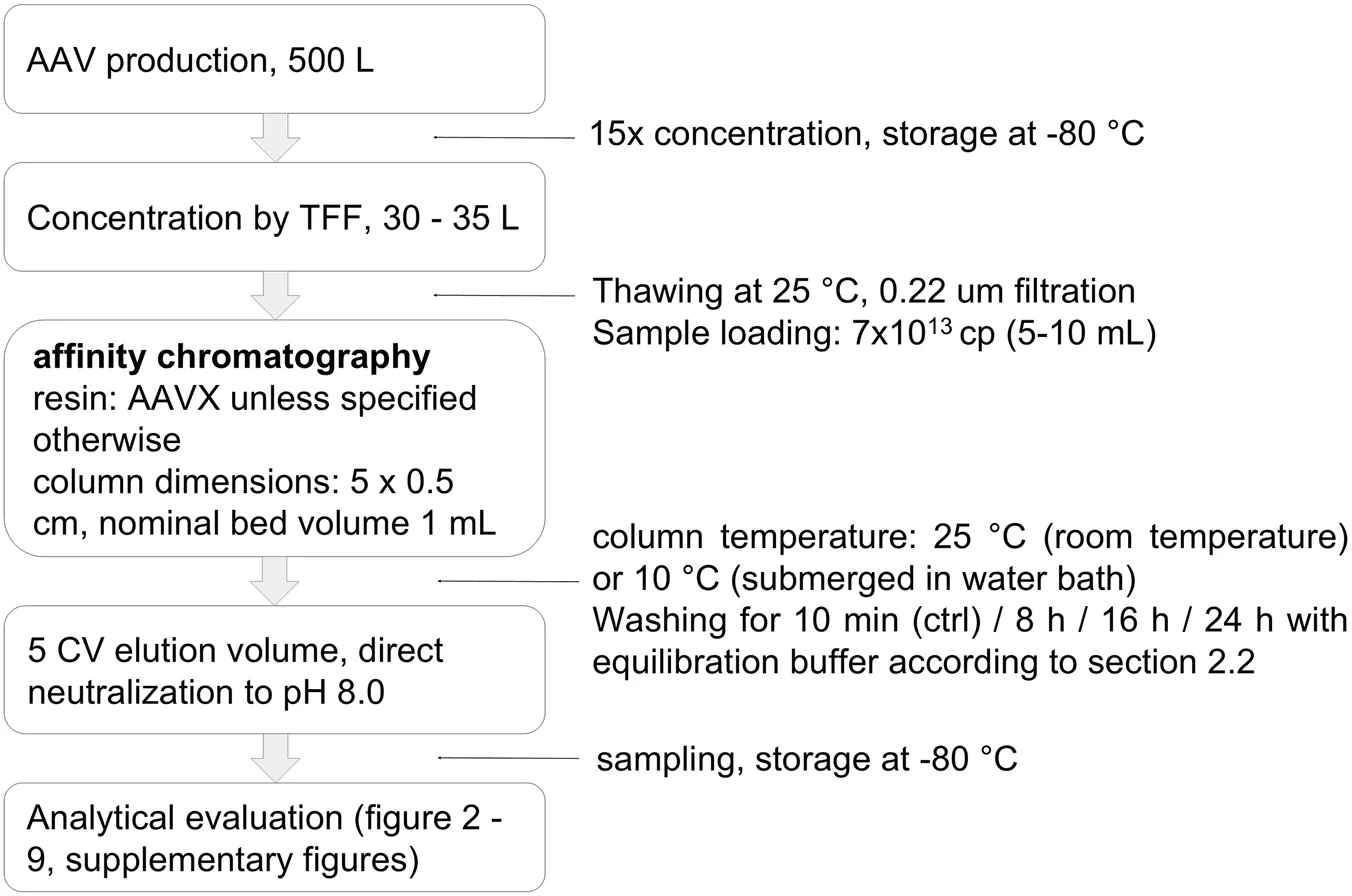
Overview of the experimental workflow. AAV harvest (500 L) was concentrated 15-fold by TFF (30–35 L) and stored at −80 °C. Following thawing (25 °C) and 0.22 µm filtration, 7 × 10^13^ capsids (5–10 mL) were loaded onto a column containing affinity resin (1 mL bed volume). Runs were performed at 10 °C or 25 °C across wash durations of 10 min (control), 8 h, 16 h or 24 h. Every elution pool (5 CV) was directly neutralized to pH 8.0 prior to −80 °C storage and analytical evaluation.

Aliquots of concentrated or unconcentrated harvested cell culture fluid were thawed in a water bath at 25 °C and filtered with a 0.22 µm Minisart® syringe filter (Sartorius AG, Göttingen, DE) prior to analysis. Chromatographic experiments were performed on an ÄKTA Avant 150 system (Cytiva). All experiments utilized prepacked columns (0.5 × 5 cm) with a nominal bed volume of 1 mL, provided by the respective manufacturers. The affinity resins evaluated in this study were POROS™ CaptureSelect™ AAVX and POROS™ CaptureSelect™ AAV9 (Thermo Fisher Scientific, Waltham, MA, USA), as well as Capto™ AVB (Cytiva).

A constant residence time of 2 min, corresponding to 0.5 mL/min or 30 column volumes (CV) / hour was used for every experiment, resulting in a constant column pressure of 0.02 MPa with the flow restrictor enabled. Prior to first use, all columns were subjected to an initial cleaning-in-place (CIP) protocol with 100 mM Glycine/Glycine-HCl, 2 M NaCl, 0.005% (v/v) Poloxamer 188 (P188) followed by 100 mM phosphoric acid and 25 mM NaOH (Supplementary Table S1). Following equilibration using 25 mM Tris/Tris-HCl, 150 mM NaCl, 2 mM MgCl_2_, 0.005% (v/v) P188, pH 7.4, the columns were loaded to a target load density of 7 × 10^13^ capsids/mL of resin, a load density well below the maximum dynamic binding capacity of any resin tested in this work (Laroudie and Bazot, 2024).

A 5 CV material chase with equilibration buffer was performed through the buffer line to ensure complete material loading. This was followed by a washing phase with equilibration buffer of variable length between 5 CV as control and 245–725 CV to achieve binding durations of 8–24 h longer than the control. Capsids were eluted using either acetic acid buffer (pH 3.5 for AAV9WT) or glycine-HCl buffer at pH 2.5 for rAAV2 and AAV8WT. Eluates were collected in tubes pre-filled with neutralization buffer at a 1:4 (v/v) ratio for adjustment to pH 8.

The material was aliquoted and stored at −80 °C for further analysis. Each chromatographic cycle concluded with a CIP step to prevent potential carry-over. Detailed information on buffer compositions and specific process parameters is provided in Supplementary Table S1.

During large-scale processing, unconcentrated cell culture fluid may be cooled with process water after lysis. During long loading phases, the chilled feed can lower the temperature of the column itself. To simulate this effect, a column was chilled within technical limitations at 10 °C for 24 h by submerging column body and column inlet in a temperature-controlled water bath (Alpha RA8, LAUDA DR. R. Wobser GmbH & Co. KG, Lauda-Königshofen, DE) whilst washing with buffer as described above. Additional cooling of buffer and load material was evaluated but showed no effect.

#### 2.2.1 Chromatography experiments with unconcentrated load

To ensure experimental comparability between the 24-h buffer wash and the loading of unconcentrated material, 12 mL of AAV8WT unconcentrated material (TFF feed; 2.34 × 10^12^ cp/mL) were diluted with the corresponding permeate (712 mL, 6 × 10^10^ cp/mL). This facilitated a continuous 24-h loading phase at a constant residence time of 2 min while maintaining the target load density. Following this loading period, the process followed the standard protocol as described above with a 5 CV wash.

### 2.3 Analytical methods

#### 2.3.1 Electroluminescence immunoassay (ECLIA)

Total capsid titers were determined by ECLIA on a cobas pro e801 device (Roche Diagnostics, Penzberg, Germany) as previously described (Bongers et al., 2026). Quantification relied on a sandwich principle using magnetic particle capture and photomultiplier detection, with titers calculated against an established calibration curve.

#### 2.3.2 Digital polymerase chain reaction (dPCR)

Viral genome titers were determined with a dPCR assay on a Qiacuity Eight system (Qiagen, Hilden, Germany) as previously described (Bongers et al., 2026). Following nuclease treatment and Proteinase K-digestion, viral DNA was quantified using the QIAcuity Probe PCR Kit with Smal digestion. Results were analyzed using the QIAcuity Software Suite v2.5.0.1, following standardized manufacturer protocols.

#### 2.3.3 Dynamic Light scattering (DLS)

Capsid size distribution and aggregation were monitored *via* DLS as previously described (Heckel et al., 2026; Cole et al., 2021; Rodenstein et al., 2025). Briefly, 30 µL aliquots were analyzed in a DynaPro® Plate Reader III (Wyatt Technology, Santa Barbara, CA, USA). The hydrodynamic radius (R_h_) and relative mass percentages were determined for monomeric species (10–100 nm) and higher-order aggregates (100–1000 nm).

#### 2.3.4 Post-translational modifications (PTMs) by liquid chromatography - mass spectrometry (LC-MS)

Samples were prepared as described previously (Heckel et al., 2026) and analyzed on a Vanquish™ Flex UHPLC (Thermo Fisher Scientific) equipped with a ACQUITY UPLC Peptide CSH C18 column (Waters Corporation, Milford, MA, USA) and coupled to an Orbitrap Exploris™ 240 mass spectrometer. Relative amounts of PTMs at specific sites were calculated with Byonics (Protein Metrics, Boston, MA, USA) and labeled according to common VP1 nomenclature.

#### 2.3.5 Quantification of non-encapsulated DNA

Non-encapsulated double-strand DNA was quantified with a QuBit™ dsDNA HS (High Sensitivity) Assay Kit. The samples were prepared by diluting the vector material in QuBit working solution at a 1:9 (v/v) ratio, followed by a 2-min incubation at room temperature. Fluorescence was measured using a QuBit™ 4 Fluorometer after calibration with the provided standards (ThermoFisher, 2022).

#### 2.3.6 Host-cell protein electroluminescence immunoassay (HCP-ECLIA)

HEK293 HCP-content was determined with an internal sandwich-type immunoassay using polyclonal antibodies. HCP content in samples was calculated from an external standard curve with known concentrations of HCPs.

#### 2.3.7 Cell-based functional transduction assay

Biological potency was evaluated with an *in vitro* transduction assay as previously described (Heckel et al., 2026); the cell line present in this study was obtained internally. Human-derived cells were seeded at 25k cells/well in a tissue culture 96 well plate (Thermo Fisher Scientific) and cultured in Dulbecco’s Modified Eagle Medium, supplemented with 10% fetal bovine serum. Transduction was normalized to the viral genome titer of each individual sample, and cells were tranduced across a serial dilution range (multiplicity of infection [MOI]: 0.6–200,000). After 24 ± 4 h, mGreenLantern (mGL) expression was quantified using a FACSLyric™ system (BD Biosciences, Franklin Lakes, NJ, USA). Relative potency was derived from the linear portion of the sigmoidal dose-response curve between the log-transformed MOI and the percentage of mGL-positive cells.

Potency assays were conducted across three independent biological experiments per condition. To account for analytical variation, each sample was analyzed in technical triplicate, and these measurements were averaged to yield a single value per biological replicate. Results are expressed as the mean ± standard deviation (SD) (*n* = 3) across the three biological replicates.

#### 2.3.8 Quantitative proteomics analysis of host cell proteins

Quantitative proteomics analysis was performed according to a protocol provided by (Viodé et al., 2026). Protein samples were reduced with 15 mM TCEP, alkylated with 55 mM CAA, and digested with trypsin (1:20 w/w) using a filter-aided sample preparation (FASP) protocol on 10-kDa centrifugal filters in the presence of 0.02% DDM. Following desalting *via* BioSPE® PurePep columns (Affinisep, Le Houlme, France), peptides were separated on an Aurora® Ultimate™ XT C18 column (IonOpticks, London, England) using a 30-min analytical gradient (Ultimate 3000 nanoHPLC; Thermo Fisher Scientific) and analyzed on an Exploris 240 mass spectrometer (Thermo Fisher Scientific) in data-independent acquisition (DIA) mode utilizing 60 sequential MS2 windows. Raw data were processed using Spectrometry (Biognysis AG, Schlieren, Switzerland) in directDIA mode against a combined *Homo sapiens* and rAAV database, applying a strict 1% false discovery rate (FDR) and requiring at least two peptides per protein for positive identification.

#### 2.3.9 Next-generation sequencing (NGS)

For next-generation sequencing the DNA was extracted from the capsids following the protocol of the QIAamp MinElute Virus Spin Kit (Qiagen). After extraction, a double strand synthesis was performed. Therefore, the genomes were incubated at 95 °C to denature any annealed genomes. The double strand synthesis was performed using a Bst 3.0 polymerase (16 units per 500 ng ssDNA) in 1× Isothermal Amplification Buffer II containing a final concentration of 8 mM MgSO4, and 1.4 mM of each dNTP in a total reaction volume of 50 μL. The synthesis was performed at a temperature of 65 °C for 1.5 h, followed by 5 min step at 80 °C. After the double-strand synthesis, the linbrary was prepared following the protocol of Pacific Biosciences (Preparing multiplexed AAV SMRTbell libraries using SMRTbell prep kit 3.0; Pacific Biosciences, Menlo Park, CA, USA) with 2x amount of the magnetic beads instead of ×1.3 as recommended by the manufacturer. The generated libraries were sequenced on a Sequel IIe instrument (Pacific Biosciences) using the adeno-associated virus application in the run and sample setup.

#### 2.3.10 Fragment Analyzer (FA)

ssDNA extraction from AAVs was done by incubating 20 μL rAAV sample with 2.5 μL of protease (Qiagen QIAamp MinElute Virus Spin Kit; Qiagen) at 56 °C for 15 min. For length determination of the extracted ssDNA a Fragment Analyzer 5300 instrument (Agilent Technologies, Santa Clara, CA, USA) was used.

### 2.4 HPLC methods

The liquid-chromatography (LC) methods described in Sections 2.4.1 and 2.4.2 were performed on an Agilent 1260 Infinity II UHPLC (Agilent). The system was equipped with a high-speed binary pump (G7120A), an online sample manager (G3167A), a multi-column compartment (G7116A), a UV diode array detector (G7117B) equipped with a 60 mm Ma-Light Cartridge Cell (4 μL; G4212-60007) and a fluorescence detector (FLD; G1321B).

#### 2.4.1 Size exclusion chromatography (SEC)

The abundance of high molecular weight (HMW) and low molecular weight (LMW) species relative to monomeric AAV capsids was quantified with SEC. Samples were diluted to a target concentration of 1 × 10^12^ capsids/mL using a potassium-based mobile phase with an organic additive. A sample volume of 10 µL was loaded on a Biozen™ 3 µm dSEC-7 column (150 × 4.6 mm; Phenomenex, Torrance, CA, USA), with temperature controlled and set to 25 °C. Separation was performed at a flow rate of 0.4 mL/min over a 15 min run time with the photo multiplier tube (PMT) gain set to 12 (λ_ex_ = 280 nm, λ_em_ = 350 nm).

#### 2.4.2 Hydrophilic interaction liquid chromatography (HILIC)

Capsid protein stoichiometry (VP1:VP2:VP3) was analyzed with HILIC, using a protocol adapted from (Liu et al., 2020). 5 μL per sample were directly injected on a ACQUITY Glycoprotein BEH Amide column (300 Å, 1.7 µm, 150 × 2.1 mm; Waters). The system was operated at 60 °C at a constant flow rate of 0.2 mL/min at a PMT gain of 12 (λ_ex_ = 280 nm, λ_em_ = 350 nm). Mobile phase A consisted of 80% (v/v) acetonitrile and 0.1% (v/v) trifluoroacetic acid (TFA) in water, mobile phase B consisted of 0.1% (v/v) TFA in water. The method started with a 1-min hold at 100% A, followed by a gradient to 90% A over the next 2 min and a subsequent gradient from 90% A to 80% A over 20 min. The column was then washed at 100% for 2 min, followed by re-equilibration at 100% A for 5 min. The VP1, VP2 and VP3 capsid proteins were identified on the chromatogram according to the elution order stablished by Liu et al.

### 2.5 Statistical evaluation

Quantitative results are expressed as the mean ± SD of three independent experiments. Normality of distribution and homogeneity of variances were confirmed for all datasets using the Shapiro-Wilk test and Levene´s test, respectively. Statistical significance was evaluated using a one-way analysis of variance (ANOVA) to compare means across different conditions. Following ANOVA, Tukey´s honest significant difference (HSD) *post hoc* test was applied to perform pairwise multiple comparisons between all groups. A *p*-value of less than 0.05 was defined as the threshold for statistical significance (*p < 0.05, **p < 0.01, ***p < 0.001, ****p < 0.0001). Regarding the recovery of viral particles, a complete mass balance could never be determined in this study. Due to large flow-through volumes caused by long washes of >8 h, capsid titers were very low. Results therefore either showed high deviations between replicates or fell below the lower limit of quantification for the respective assays. Additionally, capsids potentially eluting during the CIP step could not be reliably quantified, as the harsh CIP buffer immediately destroys AAVs. Importantly, all data with regards to titer, hydrodynamic radius (R_h_), viral protein (VP) ratio and transduction efficiency was normalized to the results of the respective control sample (indicated as “ctrl” in all respective figures) to ensure comparability across multiple experimental conditions and serotypes. While certain visualizations display only differences relative to the control for clarity, Tukey´s HSD was maintained across all datasets to ensure a consistent and rigorous statistical framework. All statistical computations and data visualizations were performed using BioRender (BioRender, Toronto, ON, Canada), utilizing R version 4.2.2.

## 3 Results and discussion

Despite recent advances (Selvaraj et al., 2020; Merten, 2024), production of rAAVs in biopharmaceutical manufacturing is challenged by low upstream titers (Nie et al., 2025), necessitating a concentration step or long loading phases over multiple hours to achieve target utilization on currently available resin beads. While potential instabilities during binding to AEX-resin have been documented (Khanal et al., 2023), the stability of rAAVs bound to affinity ligands remains largely unexplored. Understanding the implications of these prolonged capsid-ligand interactions is essential for the development of robust, scalable processes.

To address this gap, we implemented a systematic analytical framework using three serotypes (rAAV2, AAV8WT, AAV9WT) and CaptureSelect™ AAVX resin. To isolate resin-mediated degradation from instability of the load material, we performed parallel “on-bench” stability controls by holding the material on the bench for 24 h before subjecting it to purification following the same protocol as for the control. The experiments confirm that all serotypes remained stable in the loading buffer matrix for 24 h, with AAV8WT deamidation being the only exception (Section 3.6; Supplementary Figures S1-S3). This allowed us to attribute any observed shifts in CQAs specifically to capsid-ligand interaction.

To ensure experimental consistency and overcome material constraints, our primary study design utilized a simulated loading approach, wherein concentrated material was rapidly bound, followed by an extended wash phase of up to 24 h at both ambient room temperature (RT) and 10 °C. This design allowed almost identical binding durations for all capsids, opposed to a binding duration gradient caused by long loading times. The study concludes with a direct comparison of a 24-h wash with continuous direct loading to evaluate how the presence of an impurity-rich mobile phase alters the rAAV stability evaluated in wash experiments.

### 3.1 Impact of extended wash duration on vector yield

Capsid and viral genome yield of the eluted pools were assessed with ECLIA and dPCR respectively. Observed losses were found to be serotype dependent. While AAV8WT showed constant capsid yield corresponding to 7.60 × 10^12^ cp/mL in the elution phase across all experimental conditions independent of the wash duration (Figures 2C,F), both AAV9WT and rAAV2 demonstrated a significant reduction in capsid elution yield after 8 h (from 9.89 × 10^12^ cp/mL to 7.08 × 10^12^ cp/mL or 72% capsid yield and from 7.59 × 10^12^ cp/mL to 4.96 × 10^12^ cp/mL or 65% capsid yield respectively; Figures 2A,B) and viral genome elution yield (from 2.06 × 10^12^ vg/mL to 1.10 × 10^12^ vg/mL or 53% viral genome yield and from 1.48 × 10^12^ vg/mL to 9.79 × 10^11^ vg/mL or 66% viral genome yield respectively; Figures 2D,E) with no further significant decreases observed after 8 h. For all serotypes, the capsids missing from the elution phase during control experiments were not found in the flow-through and therefore remained on the column until CIP. Capsids additionally missing from the elution phase during extended hold-times were eluted during the wash and can be reloaded and bound to the resin again (data not shown).

**FIGURE 2:**
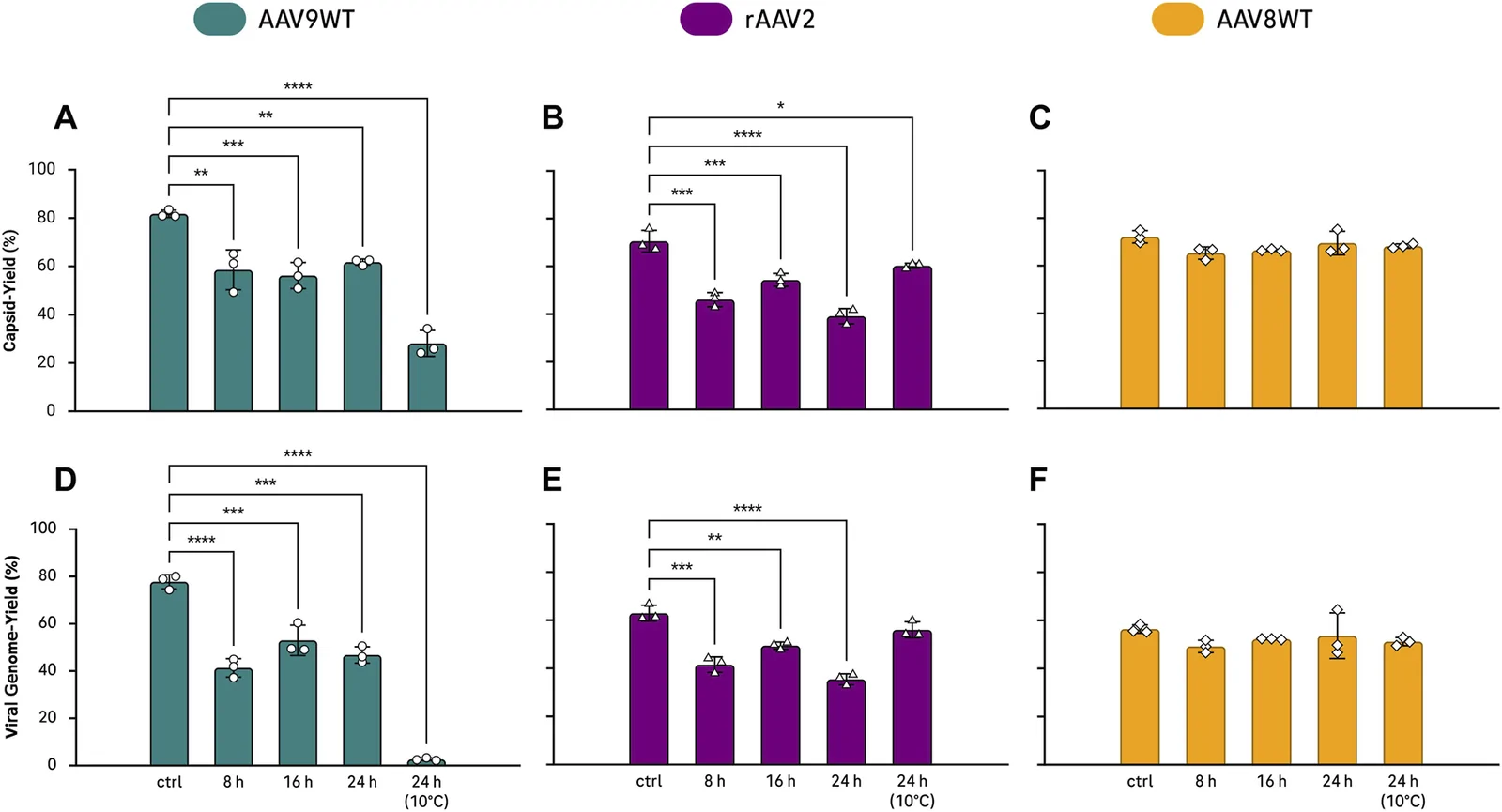
Influence of extended on column hold time and temperature on the elution yield of capsids and viral genomes. Yields were calculated following varied wash durations on AAVX affinity resin for AAV9WT **(A,D)**, rAAV2 **(B,E)** and AAV8WT **(C,F)**. Titers were determined with ECLIA and dPCR, respectively. All samples were normalized to a respective control (ctrl) with a wash duration of 5 CV/10 min. Experimental groups were subjected to extended wash phases (8 h, 16 h, 24 h) at RT with an additional cohort washed on column for 24 h at 10 °C. Data represent the mean ± SD (n = 3) and is expressed following statistical evaluation according to Section 2.5. For better readability, only significant deviations from the control are indicated. All sample groups without indicators did not show a significant deviation from the control.

Notably, 24 h of equilibration-buffer wash at 10 °C caused a 95% viral genome yield reduction to 6.6 × 10^10^ vg/mL in the elution phase for AAV9WT (Figure 2D) but had no impact on rAAV2 and AAV8WT, indicating a temperature-dependent change in binding strength unique to this serotype-ligand combination. The yield loss was higher for full than for empty capsids, resulting in a full-to-total ratio in the elution pool of less than 2%, a reduction of more than 90% from the load material with an initial full-to-total ratio of 21.9%.

### 3.2 Stoichiometric shifts in viral protein composition

The relative distribution of the three capsid proteins (VP1, VP2, VP3) was analyzed with HILIC-FLR to assess the impact of extended on-column hold-time on vector stoichiometry (Figure 3). Across all three serotypes, a consistent trend towards increased relative VP3 abundance was observed in the elution pool. This effect was especially pronounced for AAV9WT, with relative VP3 abundance reaching a maximum enrichment of approximately 1.9% after 24 h at 10 °C, compared to the respective control (Figure 3A). rAAV2 exhibited a similar behavior with a significant increase in relative VP3 of up to 1.5% after 8 h at RT with no further increase after longer wash durations (Figure 3B). In contrast, VP3 content did not change significantly at RT for AAV8WT, a statistically significant increase in relative VP3 abundance to 0.9% above the control was only observed for 24 h of wash time at 10 °C (Figure 3C).

**FIGURE 3:**
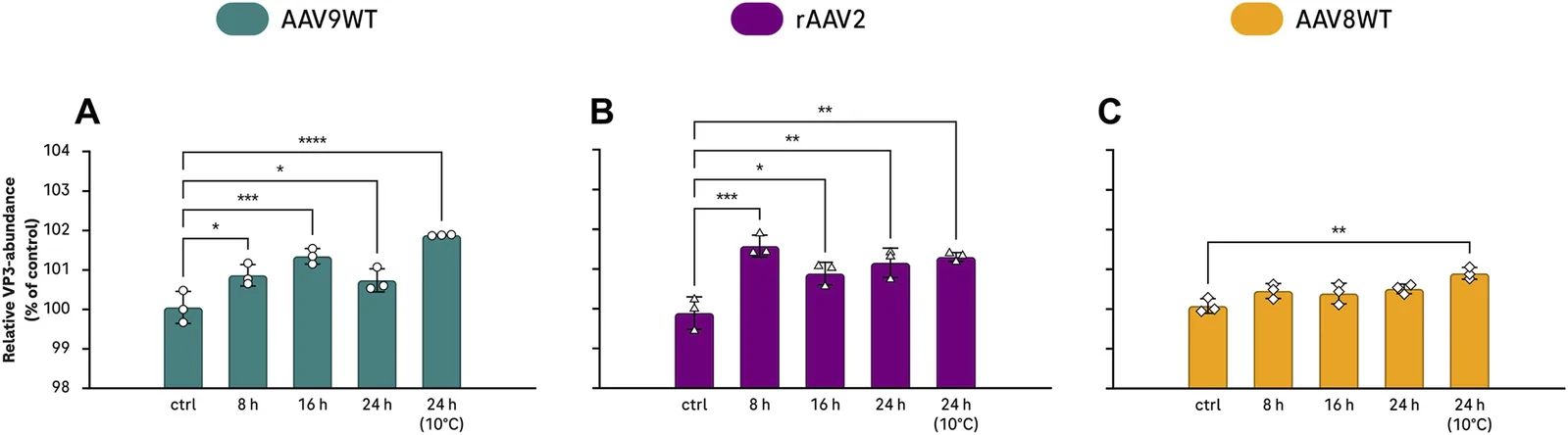
Influence of extended on column hold time and temperature on the relative abundance of VP3. VP3-content relative to a control was assessed for AAV9WT **(A)**, rAAV2 **(B)** and AAV8WT **(C)** on AAVX affinity resin. VP-ratios were measured with HILIC-FLR (λ_ex_ = 280 nm, λ_em_ = 350 nm). All samples were normalized to a respective control (ctrl) with a wash duration of 5 CV/10 min. Experimental groups were subjected to extended wash phases (8 h, 16 h, 24 h) at RT with an additional cohort washed on column for 24 h at 10 °C. Data represent the mean ± SD (n = 3) and is expressed following statistical evaluation according to Section 2.5. For better readability, only significant deviations from the control are indicated; the y-axis is truncated. All sample groups without indicators did not show a significant deviation from the control.

While VP1 and VP2 were quantified, the data is not shown as they exhibited similar trends with no significant changes between VP1 and VP2 for any condition tested, directly accounting for the relative increase in VP3.

### 3.3 Changes in aggregation and hydrodynamic radius (R_h_)

The influence of prolonged on-column hold-time on capsid aggregation, measured as the relative abundance of HMW-species by SEC-FLR, generally followed a downward trend.

For AAV9WT, a highly significant time-dependent reduction in relative HMW-abundance was observed (Figure 4A). This effect was primarily driven by the duration of the wash phase rather than temperature; HMW-content was reduced more than 5-fold from the control (0.68%) to a final value of 0.13% after 24 h for both RT and 10 °C (0.13%). A less pronounced but significant reduction was observed for AAV8WT, decreasing from 1.05% to 0.91% after 24 h at 10 °C (Figure 4C). In contrast, the rAAV2 relative HMW content showed high variability between replicates (Figure 4B). Although a marginal increase in HMW content was found to be statistically significant for the 24-h RT condition (*p* < 0.05), this was not supported by a progressive time-dependent trend. We found no differences between signals obtained by SEC-UV compared to SEC-FLR that would indicate the presence of pure DNA-peaks, the data is therefore not shown.

**FIGURE 4:**
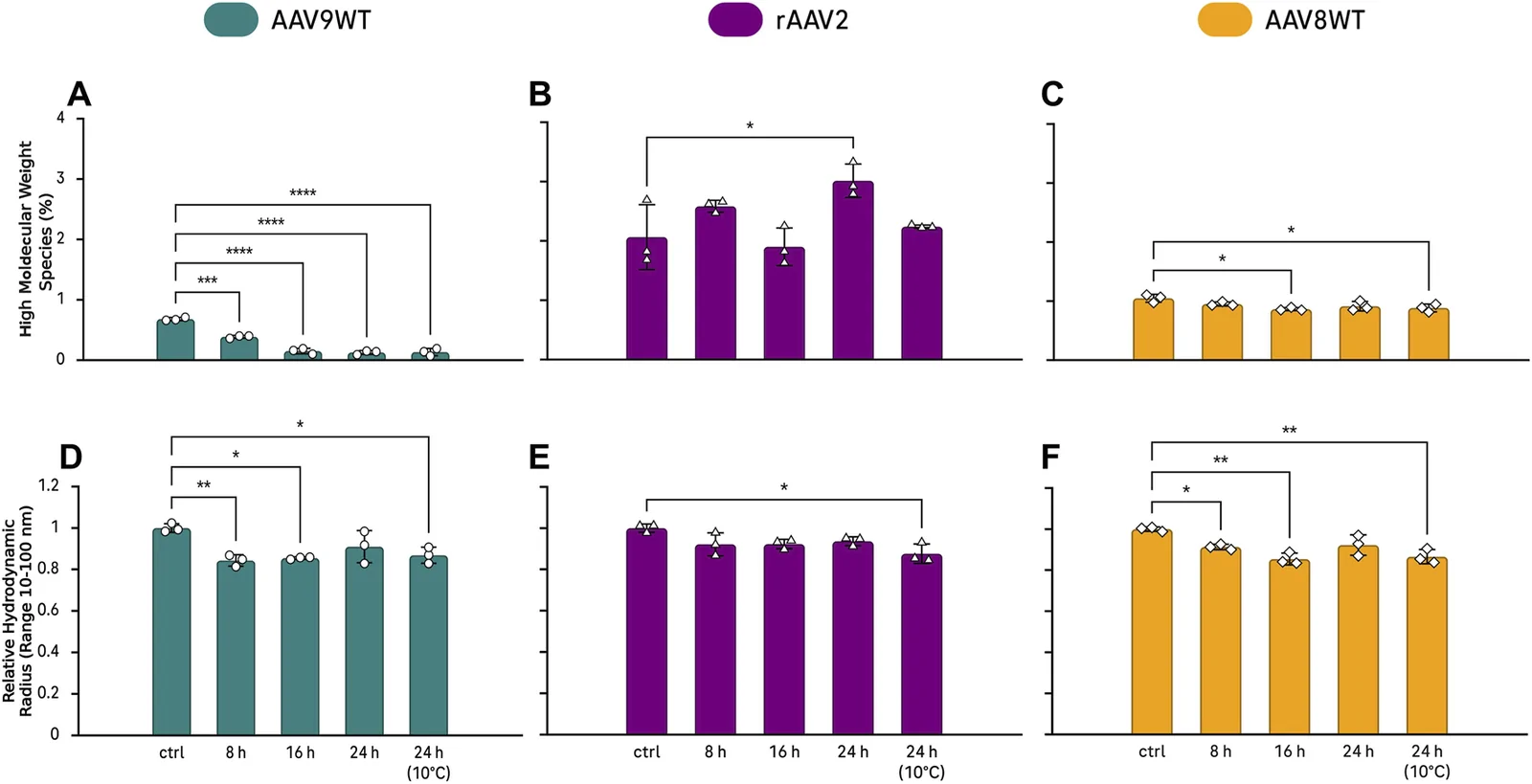
Influence of extended on column hold time and temperature on HMW-content and R_h_. Both biophysical properties were assessed for AAV9WT **(A,D)**, rAAV2 **(B,E)** and AAV8WT **(C,F)** on AAVX affinity resin. Shown is the amount of high molecular weight species relative to the respective AAV monomer peak by SEC-FLR (λ_ex_ = 280 nm, λ_em_ = 350 nm; A-C; low molecular weight species were observed but are not shown) and the normalized R_h_ in the range of 10–100 nm by DLS **(D–F)**. All samples were normalized to a respective control (ctrl) with a wash duration of 5 CV/10 min. Experimental groups were subjected to extended wash phases (8 h, 16 h, 24 h) at RT with an additional cohort washed on column for 24 h at 10 °C. Data represent the mean ± SD (n = 3) and is expressed following statistical evaluation according to Section 2.5. For better readability, only significant deviations from the control are indicated. All sample groups without indicators did not show a significant deviation from the control.

The hydrodynamic radius (R_h_), measured by DLS, followed a similar downward trend as aggregation (Figures 4D–F). Across all serotypes, the R_h_ decreased as a function of wash time rather than temperature. The normalized R_h_ for AAV9WT and AAV8WT decreased by 16% and 9% within the first 8 h, respectively, before reaching a stable plateau with no further significant changes through 24 h. For rAAV2, the shift in R_h_ was marginal across most time points, with a statistically significant decrease only noted at the 24-h 10 °C condition (Figure 4E).

### 3.4 Clearance of host-cell proteins (HCP) and non-encapsulated host-cell DNA (HCDNA)

The impact of extended on-column hold-time on the clearance of process-related impurities was evaluated by quantifying residual HCP and non-encapsulated HCDNA in the affinity eluates (Figure 5). Initial HCP concentrations in the control varied by serotype, with AAV8WT showing the highest impurity level (∼1050 ng/mL; 3.34-log removal factor), followed by rAAV2 (∼680 ng/mL; 3.34-log removal factor) and AAV9WT (280 ng/mL; 2.77-log removal factor). This agrees with findings showing that the HCP content strongly depends on upstream manufacturing conditions and can consequently differ between serotypes (Leibiger et al., 2026). Across all serotypes, extended on-column washing resulted in a significant removal of residual HCPs (Figures 5A–C). For AAV9WT and rAAV2, HCP levels dropped below the limit of quantification (LOQ) at the first timepoint investigated (8 h; Figures 5A,B), corresponding to 4.91-log removal and 4.87-log removal, respectively. AAV8WT HCP-levels remained above the LOQ with around 45 ng/mL (4.71-log removal), which was expected considering the comparably high initial HCP-concentration in the control. No temperature-dependent differences in HCP clearance were found for any serotype.

**FIGURE 5:**
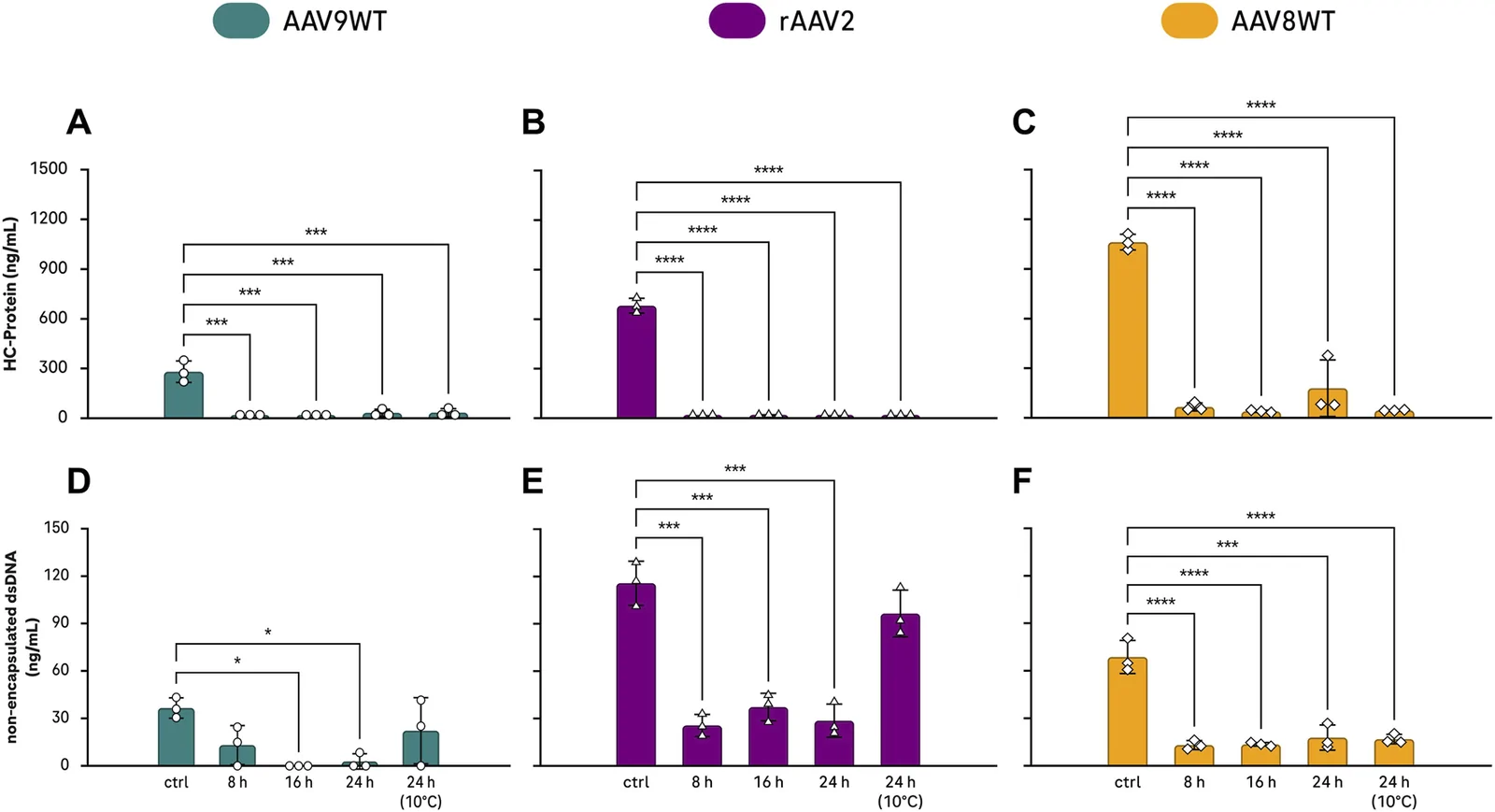
Influence of extended on column hold time and temperature on the clearance of process-related impurities. Residual concentrations were quantified for AAV9WT **(A,D)**, rAAV2 **(B,E)** and AAV8WT **(C,F)** on AAVX affinity resin. HCP and non-encapsulated dsDNA were measured by ECLIA and a fluorophore assay, respectively. Samples were subjected to a standard wash (ctrl; 5 CV/10 min), extended wash phases at RT (8 h, 16 h, 24 h), or an extended wash phase at 10 °C (24 h). Data represent the mean ± SD (n = 3). Statistical significance was determined according to Section 2.5. For better readability, only significant deviations from the control are indicated. All sample groups without indicators did not show a significant deviation from the control. Values below the limit of quantification are represented by bars at 20 ng/mL.

The clearance of HCDNA was more specific to the serotypes investigated (Figures 5D–F). DNA levels of the control ranged from ∼37 ng/mL (AAV9WT) over ∼69 ng/mL (AAV8WT) to ∼116 ng/mL (rAAV2). For AAV8WT, extended washing provided robust and consistent HCDNA clearance, with concentrations significantly reduced to <20 ng/mL across all timeframes and temperatures (Figure 5F). AAAV9WT exhibited significant HCDNA reduction after 16 and 24 h at RT (Figure 5D), rAAV2 HCDNA concentrations were reduced to ∼30 ng/mL for 8, 16 and 24 h at RT (Figure 5E). A wash at 10 °C proved less effective for HCDNA clearance for both AAV9WT and rAAV2 with no significant reduction compared to the control, with rAAV2 retaining ∼96 ng/mL of residual HCDNA (Figures 5D,E).

### 3.5 Analysis of host-cell proteins by quantitative proteomics

To gain deeper insight into the composition of the impurity profile, LC-MS-based proteomics was conducted on AAV9WT eluates. The protein landscape was compared between the control and the 24-h RT condition.

The complexity of the HCP profile was significantly reduced after the 24-h wash phase (Figure 6). In the control, a high degree of identification consistency was observed, with 2522 proteins detected across all three replicates, representing 87% of all identified proteins in that condition. The number of proteins detected across all replicates for the 24-h RT condition dropped to 288. While these shared protein identifications represent 30% of the total identifications for this condition, the overall distribution was influenced by a single outlier replicate that yielded 518 identifications. Despite this variability, the proteome consistently detected after 24 h was reduced approximately 10-fold in the amount of total HCP identifications compared to the control.

**FIGURE 6:**
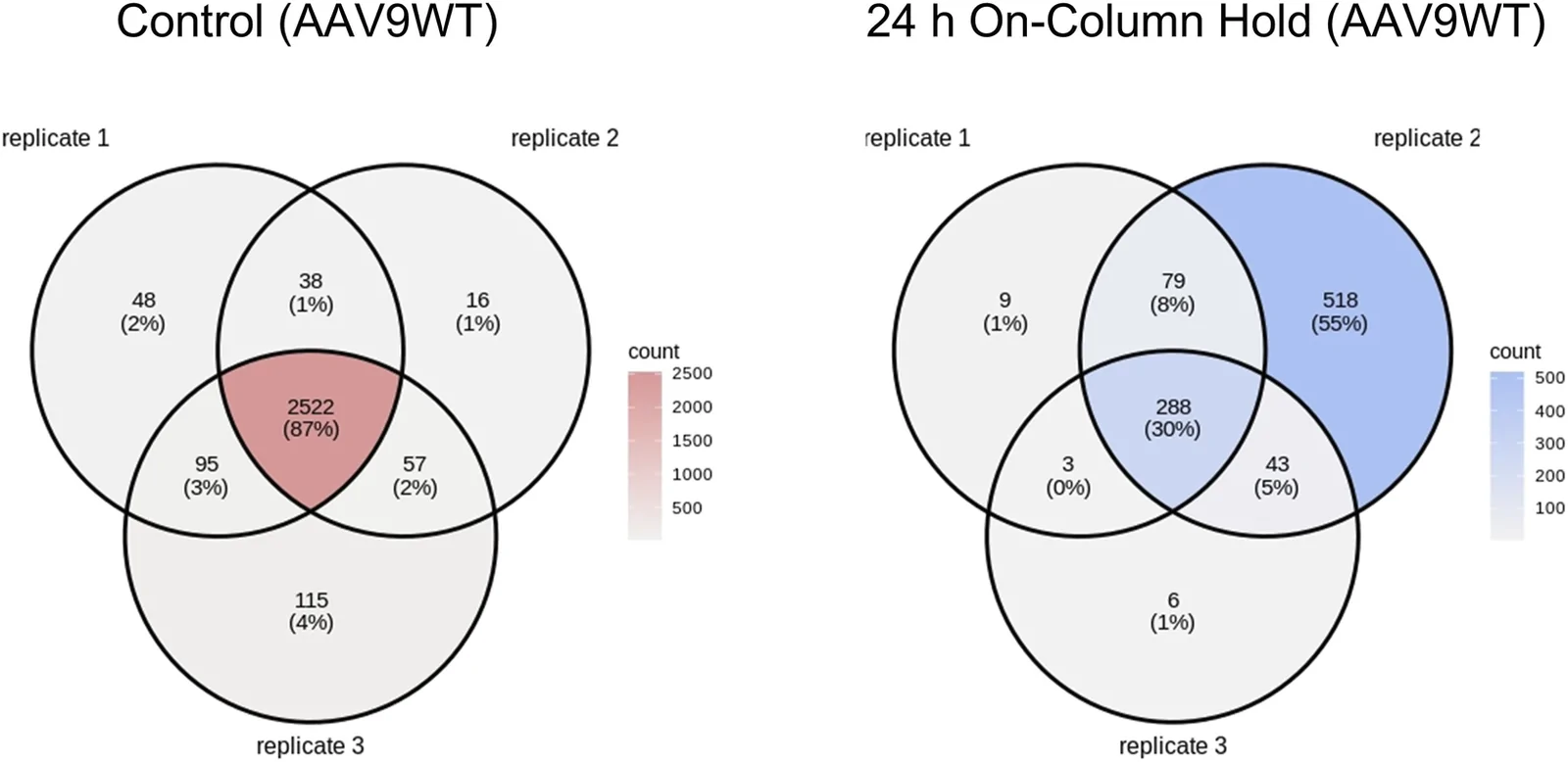
Comparative proteomic profiling of residual HCPs in AAV9WT eluates. Venn diagrams illustrate the distribution and overlap of identified HCPs across three replicates for the control (left) and the 24-h on-column wash (right). Percentages represent the fraction of protein groups relative to the total identifications within each respective experimental condition.

The most abundant HCPs found in all replicates of the control group were quantified to determine their individual reduction factors after 24 h washed on AAVX resin (Table 1). In the control, the HCP profile mainly consisted of DNA-binding protein (DNB2) and Heat shock 70 kDa protein 1A (HS71A), which were present at concentrations of 24.1 and 22.0 ng/10^12^ viral genomes (vg), respectively. Following the 24-h wash phase, these proteins were reduced to 1.1 ng/10^12^ vg and 3.6 ng/10^12^ vg, representing reduction factors of 35.8 and 10.2. DNB2 and HS71A remained the most abundant proteins, making up 5.7% and 18.2% of total HCP mass after 24 h.

**TABLE 1:** Quantitative analysis of the 10 most abundant HCPs in AAV9WT eluates and their reduction following a 24-h on-column hold, including protein abbreviation and UniProt identifier**.** Proteins are ranked by their concentration in the control. Concentrations are normalized to the viral genome tier and expressed as ng/10^12^ vg. The reduction factor was calculated as the ratio of the mean concentration in the control to that of the 24 h condition. Identification and quantification were performed by LC-MS (n = 3). Specific protein classes identified include DNA-binding proteins, histones, chaperones and metabolic enzymes.

protein	Concentration ctrl (ng/10^12^ vg)	Concentration after 24 h (ng/10^12^ vg)	Reduction factor
DNA-binding protein (DNB2, P03264)	24.08	1.12	35.82
Heat shock 70 kDA protein 1A (HS71A, P0DMV8)	21.96	3.57	10.23
Chromobox protein homolog 1 (CBX1, P83916)	3.5	0.02	275.38
Alpha-enolase (ENO1, P06733)	3.43	0.45	12.79
Histone H2B type 1-O (H2B1O, P23527)	3.17	0.33	16.04
Endoplasmic reticulum chaperone BiP (BIP, P11021)	3.06	0.31	16.42
Histone H2A type 1 (H2A1, P0C0S8)	2.53	0.33	12.79
L-lactate dehydrogenase B chain (LDHB, P00338)	2.09	0.36	9.67
Heat shock protein HSP 90-alpha (HS90B, P08238)	1.89	0.12	26.57
Tubulin alpha-1B chain (TBA1B, P68363)	1.84	0.11	27.65

Significant clearance was also observed for nuclear proteins, specifically histones H2B type 1-O (H2B1O) and H2A type 1 (H2A1). These species, which both exceeded 2.5 ng/10^12^ vg in the control, were both reduced to 0.3 ng/10^12^ vg in the 24-h on-column group. The highest relative clearance was achieved for Chromobox protein homolog 1 (CBX1), with a reduction factor of 275.4.

All other high-abundance impurities, including metabolic enzymes (ENOA, LDHB), chaperones (BIP, HS90B) and structural proteins (TBA1B), showed consistent and substantial reductions, with individual reduction factors ranging from 9.7 to 27.7. Cumulatively, the total concentration of these 10 HCPs decreased from 67.6 ng/10^12^ vg in the control to 6.7 ng/10^12^ vg after 24 h.

### 3.6 Payload integrity and transduction efficiency

To evaluate if prolonged rAAV-ligand interactions affected the biological activity of the vectors, transduction efficiency was determined with an *in-vitro* cell-based potency assay (Figure 7).

**FIGURE 7:**
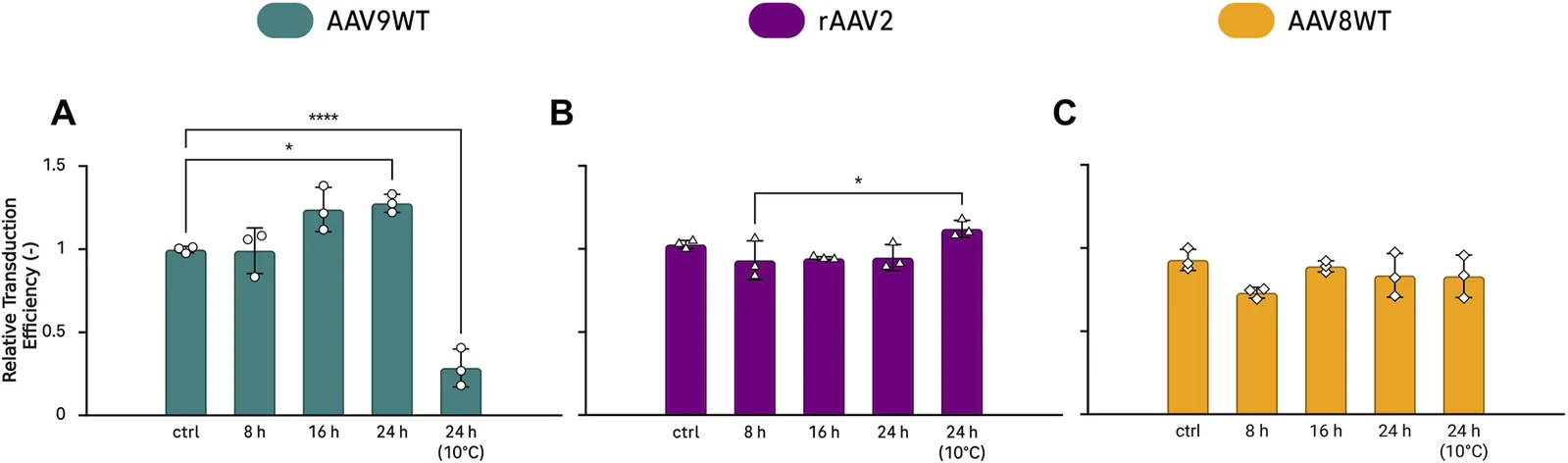
Influence of extended on column hold time and temperature on vector potency. Relative transduction efficiency was determined with a cell-based functional assay for AAV9WT **(A)**, rAAV2 **(B)**, and AAV8WT **(C)** eluates. Potency was compared between a standard wash (ctrl; 5 CV/10 min), extended wash phases at RT (8 h, 16 h, 24 h), or an extended wash phase at 10 °C (24 h). Data represent the mean ± SD (n = 3). Statistical significance was determined according to Section 2.5. For better readability, only significant deviations from the control are indicated. All sample groups without indicators did not show a significant deviation from the control.

For AAV9WT, extended hold times at RT led to a progressive and statistically significant increase in relative potency, reaching approximately 1.3-fold compared to the control after 24 h (Figure 7A). However, due to the analytical variability of the *in vitro* potency assay (Section 2.3.7), minor statistical shifts must be interpreted with caution, the ∼1.3-fold increase is unlikely to be biologically meaningful. In contrast, a large loss of biological activity was observed after 24 h at 10 °C, where transduction efficiency decreased to 0.28 relative to the control. This represents a significant drop compared to both the control and the 24-h RT sample group. As the 24 h 10 °C eluate had a full-to-total ratio of less than 2%, the large relative number of empty capsids potentially caused this reduction (Hoyle et al., 2025).

rAAV2 maintained stable potency across most conditions, with a statistically significant but biologically likely negligible increase in transduction efficiency observed only for the 24-h 10 °C condition in comparison to 8 h at RT (Figure 7B). AAV8WT exhibited the highest degree of stability, showing no significant deviations in relative transduction efficiency regardless of binding time or temperature (Figure 7C).

The transgene integrity of rAAVs was further confirmed by capillary electrophoresis and NGS (Supplementary Figures S6-S7), with no damages to the transgene being identified within the scope of the respective analytical methods (Kontogiannis et al., 2024). Additionally, LC-MS profiling confirmed that deamidation of N57 and N94 remained constant across all tested conditions except for AAV8WT, which showed increased deamidation that was also observed after 24 h on the bench and therefore not further discussed here (Supplementary Figures S4-S5). This stability is relevant as recent literature indicates that certain modifications such as the deamidations of N57 and N94 can negatively affect capsid assembly and transduction efficiency (Giles et al., 2018). In our study, increased deamidation of AAV8WT showed no significant impact on transduction efficiency (Figure 7).

### 3.7 Insights into ligand-specific differences

To determine if phenomena reported above were ligand-dependent, the performance of AAVX resin was evaluated against CSAL9 for AAV9WT and CaptoAVB for rAAV2 (Figure 8).

**FIGURE 8:**
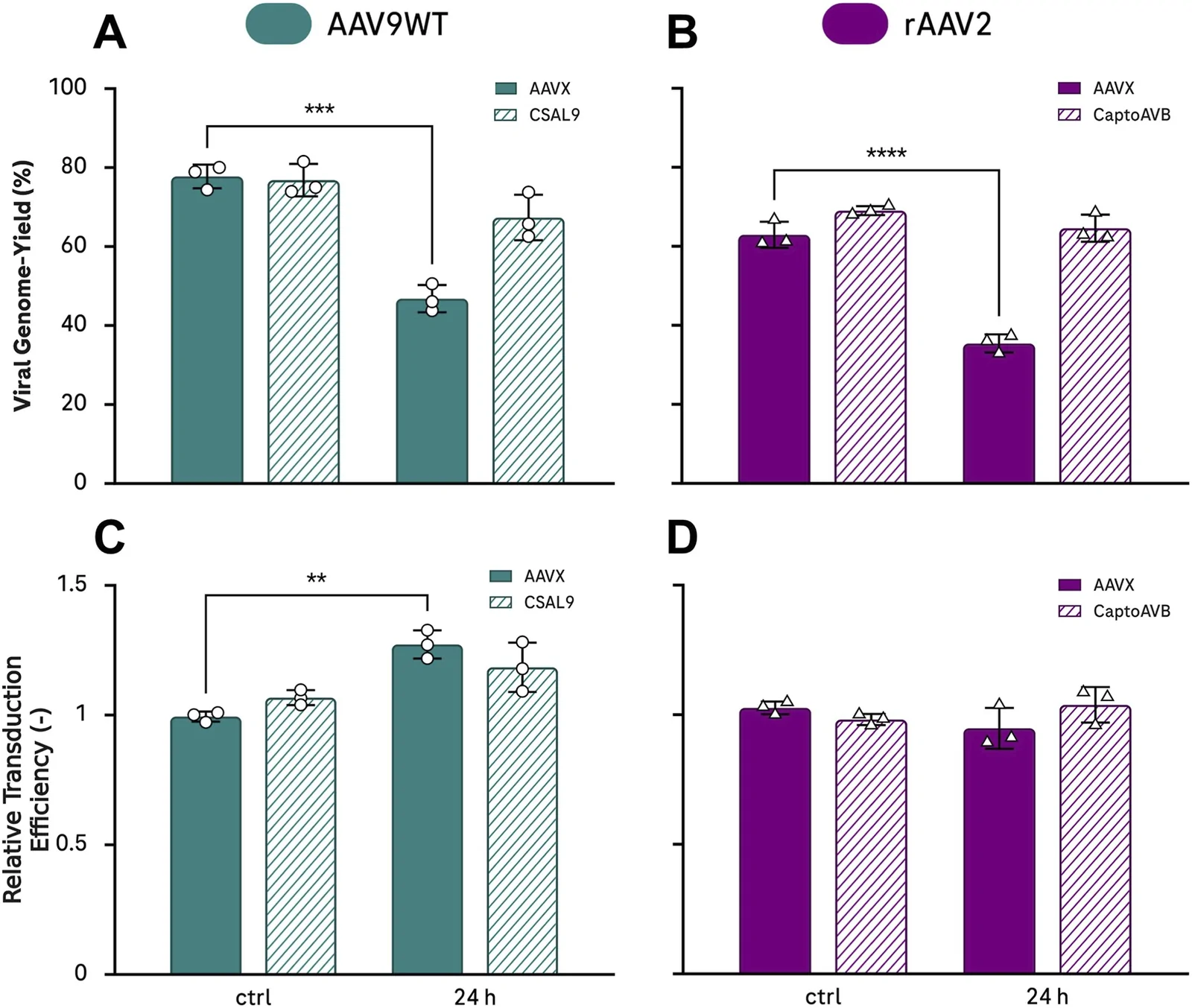
Comparative analysis of multiple affinity ligands. **(A,B)** Viral genome yield (%) and **(C,D)** relative transduction efficiency for AAV9WT (teal) and rAAV2 (purple). AAVX resin (solid bars) was compared against CSAL9 (hatched bars, teal) and CaptoAVB (hatched bars, purple), under standard conditions (ctrl; 5 CV/10 min wash) and after a 24-h wash at RT. Data represents the mean ± SD (n = 3). Statistical significance was evaluated according to Section 2.5. All sample groups without indicators did not show a significant deviation from the control.

A significant difference in elution yield was observed between the resins after a 24-h on-column hold at RT (Figures 8A,B). For both serotypes, AAVX resin exhibited a substantial drop in viral genome yield. Specifically, the AAV9WT elution yield on AAVX decreased from 78% to 47% (corresponding to a decrease in viral genome titer from 2.06 × 10^12^ vg/mL to 1.24 × 10^12^ vg/mL), while rAAV2 elution yield dropped from 63% to 35% (corresponding to a decrease in viral genome titer from 1.48 × 10^12^ vg/mL to 8.4 × 10^11^ vg/mL). In contrast, CSAL9 and CaptoAVB demonstrated significantly higher elution yield for the same experimental condition. The yield for AAV9WT on CSAL9 remained stable with a non-significant decrease from 77% to 67% (1.95 × 10^12^ vg/mL to 1.76 × 10^12^ vg/mL; Figure 8A), the yield for rAAV2 on CaptoAVB also remained stable with 69% yield in the control and 65% yield after 24 h on the column at RT (1.63 × 10^12^ vg/mL to 1.52 × 10^12^ vg/mL; Figure 8B). The relative transduction efficiency depended on the ligand (Figures 8C,D). For AAV9WT, the increase in potency after 24 h previously noted for AAVX was less pronounced on CSAL9 resin with a non-significant 1.15-fold increase (Figure 8C). For rAAV2, the transduction efficiency remained stable across both AAVX and CaptoAVB, with no significant change regardless of the 24-h hold (Figure 8D).

### 3.8 Continuous direct loading

This study serves to investigate potential rAAV degradation caused by extended binding times to camelid-derived V_H_H affinity ligands and was designed accordingly. However, a manufacturing application would not include a 24-h wash but rather long loading of harvested cell culture fluid. To bridge our results to real-life applications, we compared the previously shown standard control (ctrl) and the group subjected to a 24-h wash phase following rapid loading of highly concentrated material (24 h wash) with a condition where unconcentrated harvested cell culture fluid was loaded continuously over a 24-h period (24 h direct loading (DL)). Interestingly, while capsid yield was similar between both conditions (65%, 72%, Figure 9A), DL yielded in significantly enhanced viral genome yield (Figure 9B) of 87%, representing a substantial increase over both the control (56%) and the 24-h wash (54%). Despite these shifts in yield, transduction efficiency remained constant across all groups (Figure 9H). The relative VP3-abundance increased significantly for both conditions, with 0.5% for the 24-h wash and 1.2% for direct loading (Figure 9G).

**FIGURE 9:**
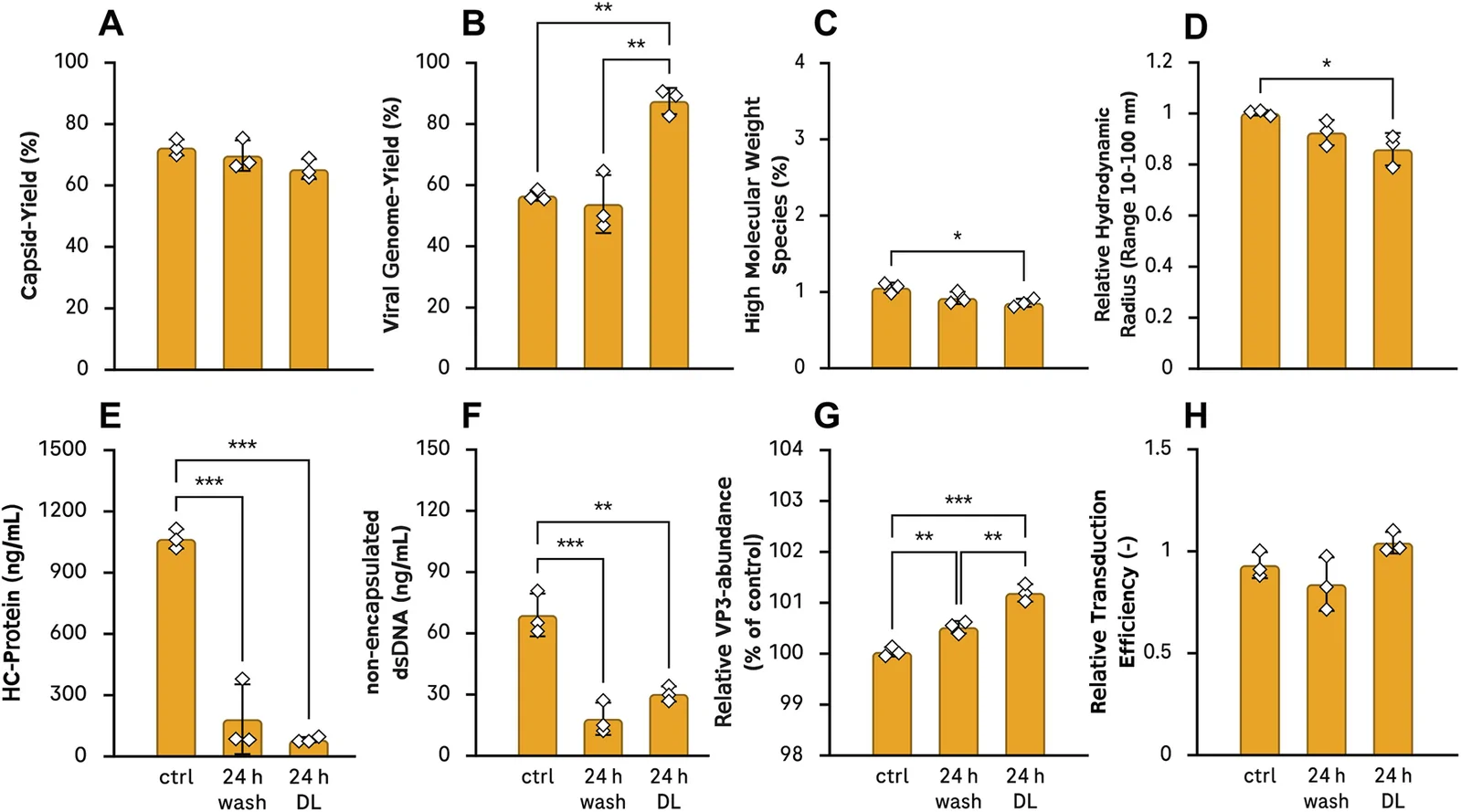
Comparison of extended on-column wash and direct loading (DL) for AAV8WT quality attributes. Evaluation of capsid yield **(A)**, viral genome yield **(B)**, HMW-species **(C)**, relative R_h_ **(D)**, residual HCP **(E)**, residual non-encapsulated HCDNA **(F)**, relative VP3-abundance **(G)** and relative transduction efficiency **(H)**. AAV8WT was purified using AAVX resin under three conditions: a control (ctrl; 5 CV/10 min), a 24-h on-column wash following rapid loading (24 h wash) and a 24-h continuous loading of unconcentrated material (24 h DL). Data represents the mean ± SD (n = 3). Statistical significance was evaluated according to Section 2.5. All sample groups without indicators did not show a significant deviation from the control.

The 24 h DL condition demonstrated a minor decrease in HMW species (from 1.0% to 0.8%; Figure 9C) and R_h_ (14% reduction; Figure 9D). The reductions do not significantly differ from the 24 h wash.

Both experimental conditions provided a reduction in process-related impurities compared to the control. HCP concentration decreased from approximately 1050 ng/mL in the control to below 200 ng/mL for the 24-h wash and below 100 ng/mL for the 24-h direct loading (Figure 9E). Similarly, non-encapsulated dsDNA was reduced from 69 ng/mL to approximately 20–30 ng/mL in both experimental groups (Figure 9F).

## 4 Discussion

This study provides a comprehensive, systematic evaluation of the effects caused by long binding durations of rAAV capsids to camelid-derived V_H_H affinity ligands. By decoupling ligand-binding mediated stress from load material instabilities, we have demonstrated that extended hold times induce significant, serotype-dependent alterations in vector elution yield, viral protein stoichiometry and impurity clearance, while transgene integrity is maintained.

CaptureSelect™ AAVX resin successfully cleared process-related impurities but introduced trade-offs. Extended hold times resulted in elution yield losses for AAV9WT and rAAV2 (Figure 2). The preferential loss of full capsids aligns with the previously shown ability of affinity ligands to exhibit serotype-agnostic selectivity between full and empty capsids, with lower affinity displayed towards full capsids (Bongers et al., 2026). However, no serotype-specific differences in yield loss were expected, as AAVX-ligands bind to the five-fold region, which is highly conserved across different clades (Mietzsch et al., 2024). In a previous work, the same three serotypes were compared for the pH necessary for elution from AAVX, finding that rAAV2 requires a lower elution pH than AAV8WT (Bongers et al., 2026), potentially caused by stronger clustering of hydrophobic charge patches on the surface (Heldt et al., 2023). Since yield losses were observed for rAAV2 but not for AAV8WT, the acidity required for capsid elution does not correlate with the binding affinity. It is to be noted that we did not vary the loading density within this study to ensure experimental consistency but suggest rigorous testing in future studies to determine potential effects on the process yield.

A substantial yield loss for AAV9WT at 10 °C (Figures 2A,D) has been observed before by Florea et al. with a cooled HPLC-system (Florea et al., 2023). They report yield losses of 40%–50%, while we show a capsid yield loss of 70%, with the difference likely caused by the longer wash duration at the same temperature. Although our study clearly demonstrates a serotype-specific elution yield loss for AAV9WT on AAVX resin following extended holds at 10 °C, defining the mechanism driving this dissociation is an area for future exploration. We hypothesize that the dissociation is linked to the variable region II (VR-II). Structural studies demonstrated that the AAVX ligands complementary-determining regions (CDRs) interact primarily with the peptide backbone of the DE and HI loops (Mietzsch et al., 2024; DiMattia et al., 2012). Because VR-II is located on the highly exposed DE-loop, we propose that lowering the column temperature to 10 °C alters the conformational flexibility of VR-II, which in turn destabilizes the AAVX binding affinity and drives capsid dissociation. Alternatively, the observed effect could simply be attributed to weaker hydrophobic interactions at lower temperatures; however, a distinct serotype-specific variation must be involved as this phenomenon was exclusively observed for AAV9WT and no other capsid tested. While directly validating a localized structural rearrangement experimentally was beyond the scope of this work, future investigations could employ Hydrogen-Deuterium Exchange Mass Spectrometry (HDX-MS) to test this theory (Ye et al., 2025; Ye et al., 2024).

Long washes provided a process advantage by systematically clearing host cell impurities (Figures 5, 6; Table 1). Several of the most abundant proteins coeluting with AAV9WT after affinity chromatography with AAVX-resin were found to be associated with DNA (DNB2, CBX1, H2B1O, H2A1). This agrees with recent work showing that proteins which interact with nucleic acids can be commonly found after affinity chromatography for rAAVs. While the same subset of proteins has above average clearance during AEX-chromatography, they are still found in drug substance (Bohlig et al., 2026). A higher clearance during affinity chromatography therefore directly yields in higher final product quality. Interestingly, non-encapsulated HCDNA was found to have a very similar reduction factor (13.1) to Histones H2A1 (12.8) and H2B1O (16.0), as also reported by Bohlig et al. (Bohlig et al., 2026). Similar to chromatin-mediated hitchhiking interactions between monoclonal antibodies and HCPs during Protein A chromatography (Gagnon et al., 2014; Shukla and Hinckley, 2008; Velde et al., 2020), DNA-histone complex likely associate with the AAV capsids, explaining both their co-elution and the observed correlation between impurity clearance and R_h_-reduction (Figure 10). Following the basic assumption that lower input equals lower output, our study demonstrates that a cleaner drug substance can be achieved by simply increasing the wash time during affinity chromatography. The next logical step is to define an economically viable wash length with minimal product loss. Due to the significantly lower impurity concentrations after 8 h it is foreseeable that significant clearance can already be achieved at much shorter timeframes.

**FIGURE 10:**
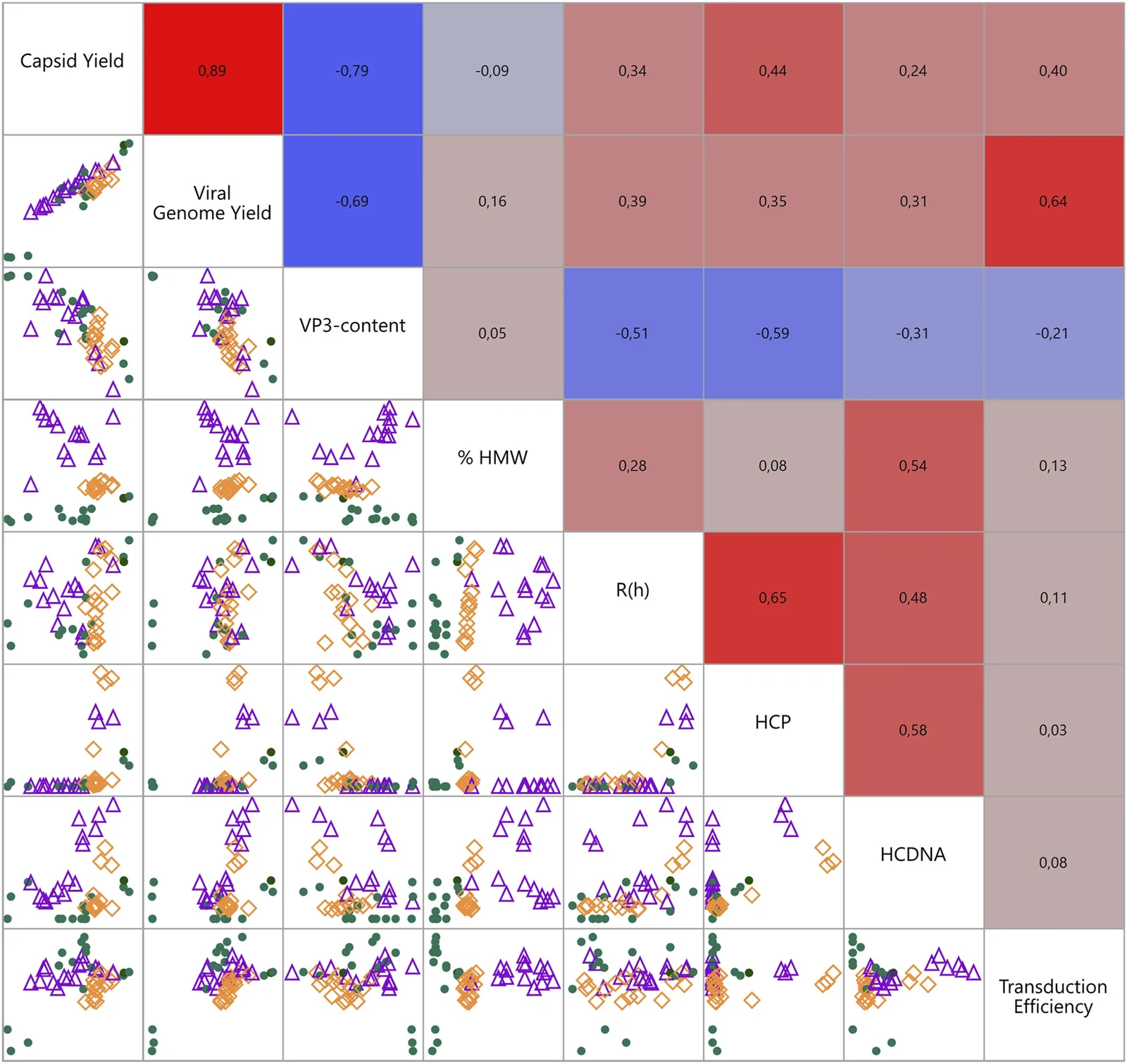
Multivariate correlation analysis of AAV quality attributes. The correlation matrix displays bivariate scatter plots in the lower triangle, illustrating the relationships between tested parameters across the three tested serotypes (AAV9WT: green circle; rAAV2: purple triangle; AAV8WT: orange diamond). The upper triangle presents a heatmap of the corresponding Pearson correlation coefficients (r), where red indicates positive and blue indicates negative correlations, with color intensity reflecting the strength of the relationship. Parameters evaluated include yield (capsid and viral genome), capsid composition (VP3-content), aggregation (% HMW), hydrodynamic size (R_h_), process-related impurities (HCP and HCDNA) and potency (transduction efficiency). Statistical significance (p-values) for each interaction is listed in Supplementary Table S2. All datapoints have been introduced in Figures 2–7.

We furthermore observed that effects on yield loss and transduction efficiency were ligand-specific rather than universal (Figure 8). Alternative ligands, specifically CSAL9 (AAV9WT) and CaptoAVB (rAAV2), successfully mitigated yield losses at constant biological potency while matching the impurity clearance of AAVX (data not shown). Structural characterizations demonstrate that AAVX and CaptoAVB are highly similar (Mietzsch et al., 2024). The CaptoAVB nanobody interacts primarily with the C-terminal residues of the HI-loop (Mietzsch et al., 2020). Similarly, high-resolution cryo-EM shows that the AAVX ligand relies on its CDR2 and CDR3 to interact with both the DE and HI loops, forming up to 16 potential hydrogen bonds per nanobody. While AAVX binds on average with two nanobodies per capsid, CaptoAVB binds with five (Mietzsch et al., 2024). Given this near-identical binding orientation, the distinct ability of CaptoAVB to prevent rAAV2 yield losses over a 24 h hold while AAVX fails to do so was unexpected. The difference likely stems from minor sequence variations within the ligands or subtle conformational differences, allowing CaptoAVB to bind with more ligands per capsid than AAVX. We hypothesize that this increased number of bound ligands per capsid provides higher structural stability, preventing the dissociation observed with AAVX over long binding times.

Cryo-EM has shown that, while both AAVX and CSAL9 target the 5-fold symmetry region, they adopt fundamentally different binding conformations. AAVX leans toward the 5-fold axis and only binds residues within a single viral asymmetry unit. In contrast, while the CDR of CSAL9 contacts the DE-loop, it spreads away from the 5-fold axis and spans across two separate viral asymmetry units (Mietzsch et al., 2020). While the exact biochemical reasons cannot be elucidated here, these structural differences provide a mechanistic explanation for the higher capsid yield of CSAL9.

As both CSAL9 and CaptoAVB resin successfully mitigated the elution yield losses observed with AAVX while fully maintaining transduction efficiency, impurity clearance and transgene integrity (data not shown), a longer wash may be encouraged for these serotype-ligand combinations to increase product quality.

Finally, we demonstrated that, within our study, a continuous direct loading approach did not negatively impact yield or other CQAs while providing high levels of impurity clearance (Figure 9). Surprisingly, full capsid yield after 24 h of direct loading significantly exceeded the yield observed in the 24-h wash and the control. We hypothesize that, as reported previously for an rAAV (Khanal et al., 2023), the utilized AAV8WT full capsids had a higher VP3-content than empty capsids, leading to stronger retention of full capsids as presented in Section 3.2 and discussed in detail below (Section 4.1). An additional cause can be the different binding equilibrium, as the mobile phase during direct loading constantly contains AAV capsids, as opposed to a wash with buffer. The VP-ratio is known to vary between serotypes (Wörner et al., 2021); changes in VP-ratio between empty and full capsids are not always observed (Heldt et al., 2024). However, molecular dynamics simulations suggest that smaller numbers of VP1/VP2 make transgene loading energetically more favorable (Parambathu et al., 2026). The VP-ratio can alter the surface chemistry (Section 4.1); therefore, every method of purification might introduce a bias towards VP-ratios. We therefore did not attempt to experimentally determine the VP3-content of pure full and empty AAV8WT capsid pools used in this project.

### 4.1 Serotype-agnostic multivariate correlation analysis of quality attributes

To evaluate serotype-agnostic correlations between different CQAs, a multivariate correlation analysis was performed across the pooled datasets of all three serotypes (Figure 10). The resulting correlation matrix presents Pearson correlation coefficients (*r*) in the upper triangle and corresponding bivariate scatter plots in the lower triangle, with statistical significance summarized in the accompanying *p*-value table (Supplementary Table S2).

A significant negative correlation was identified between relative VP3 content (Figure 3) and both capsid yield (r = - 0.79, p < 0.0001) and viral genome yield (r = - 0.69, p < 0.0001; Figure 2). These results demonstrate that a reduction in elution yield corresponds to an increase in the relative percentage of the VP3-subunit within the recovered population in the elution pool. The strong negative correlation suggests that the observed washing-off of capsids was not random but selective for subpopulations with higher relative VP1 and VP2 content. As the capsids were able to bind again upon reloading onto the same resin, this process is likely driven by dissociation rather than irreversible capsid degradation. Given that the unique N-termini of VP1 and VP2 are known to externalize through the 5-fold pores (Kronenberg et al., 2005), one might hypothesize that long binding times trigger this externalization, thereby actively displacing the AAVX ligand by direct steric hindrance. However, this mechanism is highly unlikely under the specific process conditions employed. The externalization of the VP1/VP2 N-termini is dependent on specific environmental triggers, most notably the acidic pH (pH 4.0–5.5) encountered during endosomal/lysosomal trafficking (Penzes et al., 2021). Because the 24-h on-column washes occurred at a neutral pH of 7.4, the N-termini are expected to have remained internalized within the capsid. Instead of active displacement, the selective dissociation is likely driven by steric constraints and altered surface topology resulting from a higher absolute amount of VP1/2 within the capsid (Heldt et al., 2024; Parambathu et al., 2026). Miller et al. were able to experimentally display increased structural heterogeneity in AAV9WT with different compositions of VP1/2/3 opposed to VP3-only capsids (Miller et al., 2025). As we demonstrate that yield losses occur over time, we reason that most conformers bind to the ligand initially but have reduced affinity compared to VP3-rich variants. Consequently, the continuous wash phase promoted the dissociation of VP1/2-rich capsids, effectively polishing the population towards a more homogenous VP3-dominant profile.

A long wash induced selectivity within the capsid population and, simultaneously, caused clearance of process related impurities. Both HCP and non-encapsulated HCDNA were reduced significantly compared to standard processes (Figure 5). This was directly reflected in a reduction of the R_h_ (Figures 4D–F). While it has been previously reported that full and empty capsids can exhibit differences in R_h_ (Sarmah and Husson, 2024), our multivariate analysis (Figure 10) confirms that within this study, alterations in R_h_ were primarily dictated by the residual burden of HCP (r = 0.65, p < 0.0001) and HCDNA (r = 0.48, p = 0.0008), whereas the full-to-total ratio remained constant. Coeluting impurities likely remained associated with the capsids surface, thereby slightly increasing the R_h_.

The concentration of HMW species relative to monomeric AAVs (Figures 4A–C) showed a significant positive correlation with HCDNA (r = 0.54, p = 0.0002). No such relationship was identified between HMW content and HCP concentration (r = 0.08), leading us to propose a “sticky hand” mechanism where long DNA backbones bridge capsids to form reversible aggregates. While we acknowledge that this observation is correlative within the context of our study and further validation experiments were not in scope of this work, our hypothesis is grounded in established literature.

Previous research has clearly identified residual surface-associated nucleic acids as a primary contributing factor to AAV particle aggregation (Wright et al., 2005). The presence of free or released DNA in solution has been shown to strongly alter capsid aggregation kinetics, likely due to stabilizing effects between the DNA and the exterior of the capsids (Motabar et al., 2025). Therefore, while our specific dataset only provides correlative evidence, it supports the conclusion that a “sticky hand” effect could facilitate the formation of reversible, DNA-mediated aggregates, which themselves were reduced as a function of wash duration.

Transduction efficiency showed a strong positive correlation with viral genome yield (r = 0.64, p < 0.0001). Notably, this correlation was observed despite the transduction with uniform MOI across all samples in the cell-based assay. Beyond overall viral genome yield, biological potency showed no other significant correlations. As a cell-based transduction assay is a readout of cell entry, trafficking, capsid uncoating, genome integrity and expression, its general independence from other singular analytical methods is consistent with previous observations (Kontogiannis et al., 2024; Aronson et al., 2020).

Ultimately, our results show that strategic ligand selection is essential to maximize vector yield and maintain CQAs during long hold times of AAVs on camelid-derived V_H_H-affinity ligands. Yield loss observed with AAVX affinity ligands was found to be selective for subpopulations with higher relative VP1 and VP2 content, suggesting a kinetic selection mechanism driven by local surface charge heterogeneity. Simultaneously, extended binding times provided a process advantage by systematically clearing host cell impurities, including DNA-interacting proteins such as histones H2A1 and H2B1O. Importantly, transgene integrity and transduction efficiency was maintained regardless of the capsid-ligand interaction time across all evaluated serotypes. We show that continuous loading instead of prior concentration can be a favorable mode of operation for rAAV manufacturing but needs to be evaluated for each specific serotype-ligand combination.

## Data Availability

The datasets presented in this article are not readily available because the authors do not have permission to share data. Requests to access the datasets should be directed to andres.martinez.am5@roche.com.
