# Supplementary material for: Assessing AAV stability on affinity resins and the effects of long contact times through extensive washing
Source: Front Bioeng Biotechnol. 2026 Oct 30;14:1954356. doi: 10.3389/fbioe.2026.1954356 (PMC13761220; doi:10.3389/fbioe.2026.1954356)
Supplement: Supplementary file 1 [file Supplementaryfile1.docx]

**Supplementary Material**

**Supplementary Table 1:** **Chromatographic method parameters for the affinity capture of rAAVs.** The protocol outlines standard flow rates and volumes for all steps, highlighting the variable extended wash durations (10 to 1450 min) evaluated for capsid stability. All buffers are detailed below.

| **Step** | **Column Volumes (CV)** | **Flow Rate (CV h^-1^)** | **Step Length (min)** |
| --- | --- | --- | --- |
| **Equilibration** | **5** | **30** | **10** |
| **Load** | **serotype-dependent** | **30** | **serotype-dependent** |
| **Buffer Chase** | **5** | **30** | **10** |
| **Wash** | **5 / 245 / 485 / 725** | **30** | **10 / 490 / 970 / 1450** |
| **Elution** | **5** | **30** | **10** |
| **CIP1** | **5** | **30** | **10** |
| **CIP2** | **5** | **30** | **10** |
| **CIP3** | **5** | **30** | **10** |
| **Equilibration** | **5** | **30** | **10** |

**Equilibration / Buffer Chase / Wash: 25 mM Tris / Tris-HCl, 150 mM NaCl, 2 mM MgCl_2_, 0.005 % (v/v) Poloxamer 188 (P188), pH 7.4**

**Elution (rAAV2, AAV8WT): 100 mM Glycine / Glycine-HCl, 100 mM NaCl, 2 mM MgCl_2_, 1 % (w/v) Sorbitol, 0.005 % (v/v) P188, pH 2.5**

**Elution (AAV9WT): 100 mM Acetate, 2 mM MgCl_2_, 1 % (w/v) Sorbitol, 0.005 % (v/v) P188, pH 3.5**

**CIP1: 100 mM Glycine / Glycine-HCl, 2 M NaCl, pH 1.5**

**CIP2: 100 mM Phosphoric Acid**

**CIP3: 25 mM NaOH**

**Supplementary Table 2: Statistical significance matrix (p-values) for the multivariate correlation analysis.** The table provides the calculated p-values for the Pearson correlation coefficients (r) presented in Figure 10. Values describe the significance of the linear relationship between quality attributes of AAV8WT, AAV9WT and rAAV2.

|  | **Capsid Yield** | **Viral Genome Yield** | **VP3-content** | **% HMW** | **R(h)** | **HCP** | **HCDNA** | **Transduction Efficiency** |
| --- | --- | --- | --- | --- | --- | --- | --- | --- |
| Capsid Yield | <,0001 | <,0001 | <,0001 | 0,5539 | 0,0209 | 0,0024 | 0,1157 | 0,0065 |
| Viral Genome Yield | <,0001 | <,0001 | <,0001 | 0,2876 | 0,0076 | 0,0176 | 0,0387 | <,0001 |
| VP3-content | <,0001 | <,0001 | <,0001 | 0,7258 | 0,0003 | <,0001 | 0,0414 | 0,1617 |
| % HMW | 0,5539 | 0,2876 | 0,7258 | <,0001 | 0,0621 | 0,6151 | 0,0002 | 0,3858 |
| R(h) | 0,0209 | 0,0076 | 0,0003 | 0,0621 | <,0001 | <,0001 | 0,0008 | 0,4537 |
| HCP | 0,0024 | 0,0176 | <,0001 | 0,6151 | <,0001 | <,0001 | <,0001 | 0,8244 |
| HCDNA | 0,1157 | 0,0387 | 0,0414 | 0,0002 | 0,0008 | <,0001 | <,0001 | 0,5826 |
| Transduction Efficiency | 0,0065 | <,0001 | 0,1617 | 0,3858 | 0,4537 | 0,8244 | 0,5826 | <,0001 |

**Supplementary Figure 1: AAV9WT stability during a 24-hour benchtop hold.** To isolate the specific effects of affinity ligand binding from potential load instabilities, samples were subjected to a static 24-hour hold at room temperature (24 h on bench) and compared to immediate processing (ctrl). The hold on bench induced no significant differences (ns) in (A) capsid yield, (B) viral genome yield, (C) abundance of HMW-species, (D) hydrodynamic radius, (E) HCP-burden, (F) non-encapsulated dsDNA levels, (G) VP3-abundance or (H) transduction efficiency. Data are presented as mean $\pm$ SD (n = 3). Statistical significance was determined according to section 2.4.

**Supplementary Figure 2:** **rAAV2 stability during a 24-hour benchtop hold.** To isolate the specific effects of affinity ligand binding from potential load instabilities, samples were subjected to a static 24-hour hold at room temperature (24 h on bench) and compared to immediate processing (ctrl). The hold on bench induced no significant differences (ns) in (A) capsid yield, (B) viral genome yield, (C) abundance of HMW-species, (D) hydrodynamic radius, (E) HCP-burden, (F) non-encapsulated dsDNA levels, (G) VP3-abundance or (H) transduction efficiency. Data are presented as mean $\pm$ SD (n = 3). Statistical significance was determined according to section 2.4.

**Supplementary Figure 3:** **AAV8WT stability during a 24-hour benchtop hold.** To isolate the specific effects of affinity ligand binding from potential load instabilities, samples were subjected to a static 24-hour hold at room temperature (24 h on bench) and compared to immediate processing (ctrl). The hold on bench induced no significant differences (ns) in (A) capsid yield, (B) viral genome yield, (C) abundance of HMW-species, (D) hydrodynamic radius, (E) HCP-burden, (F) non-encapsulated dsDNA levels, (G) VP3-abundance or (H) transduction efficiency. Data are presented as mean $\pm$ SD (n = 3). Statistical significance was determined according to section 2.4.

**Supplementary Figure 4:** **Changes in post-translational modifications during a 24-hour benchtop hold.** To isolate the specific effects of affinity ligand binding from potential load instabilities, samples were subjected to a static 24-hour hold at room temperature (24 h on bench) and compared to immediate processing (ctrl). Site-specific deamidation percentages at asparagine residues N57 (A-C) and N94 (D-F) were quantified with LC-MS for AAV9WT (A, D), rAAV2 (B, E) and AAV8WT (C, F). No significant changes (ns) were observed AAV9WT or rAAV2. A small but significant increase in deamidation was detected for AAV8WT at both sites (*, p < 0.05). Data represent mean $\pm$ SD (n = 3), statistical significance was determined according to section 2.4.

**Supplementary Figure 5: Influence of extended on column hold time and temperature on post-translational modifications.** Percentages of deamidation at residues N57 (A-C) and N94 (D-F) are shown for AAV9WT (A, D), rAAV2 (B, E) and AAV8WT (C, F). Experimental groups were subjected to extended wash phases (8 h, 16 h, 24 h) at RT with an additional cohort washed on column for 24 h at 10 °C. Data represent the mean $\pm$ SD (n = 3) and is expressed following statistical evaluation according to section 2.4. For better readability, only significant deviations from the control are indicated. All sample groups without indicators did not show a significant deviation from the control.

AAV9WT showed no changes in deamidation over any binding duration at any temperature tested (Supplementary Figure 5A, D). rAAV2 showed a significant decrease in deamidation at N94 after 24 hours on column at 10 °C (Supplementary Figure 5E) and a non-significant decrease in deamidation at N57 (Supplementary Figure 5B). On column holds at room temperature did not show any deviation from the control. While AAV8WT did show a significant increase in deamidation at both sites after extended binding time to AAVX (Supplementary Figure 5 C, F), it also showed increased deamidation after a 24 hour hold of the load material on the bench (Supplementary Figure 4C, F). This observation can therefore not be attributed to on-column binding for an extended period and is not further discussed in this work.

**Supplementary Figure 6: Next-generation sequencing (NGS) read length distribution of encapsulated viral genomes.** Density plots depict the length distribution of primary aligned fill-up reads for AAV9WT (A, D), rAAV2 (B, E) and AAV8WT (C, F). Serotype-specific controls (top row; 5 CV/10 min) were compared against samples subjected to a 24-hour on-column hold (bottom row) to assess transgene stability. Data was filtered to display only primary aligned reads shorter than 15 kbp. Because double-stranded intermediates are sequenced, the displayed read lengths have been divided by two to accurately represent the length of the original packaged ssDNA genome. The peaks confirm that binding durations of AAV capsids on AAVX do not induce measurable genome instabilities across any of the tested serotypes.

**Supplementary Figure 7: Capillary electrophoresis profiles of AAV transgenes.** Fragment Analyzer experiments assessed the structural integrity of the ssDNA transgene. Serotype-specific controls (top row; 5 CV/10 min) were compared against samples subjected to a 24-hour on-column hold (bottom row) for AAV9WT (A, D), rAAV2 (B, E) and AAV8WT (C, F). In each trace, the relative fluorescence units (RFU) are shown, with the left peak representing the internal lower marker and the right peak corresponding to the full-length transgene. The consistent peak morphology and absence of low-molecular-weight fragments in the 24 hour samples confirm that long on column binding does not induce genomic degradation across any of the tested serotypes.
